# Protein metalation in biology

**DOI:** 10.1016/j.cbpa.2021.102095

**Published:** 2022-02

**Authors:** Andrew W. Foster, Tessa R. Young, Peter T. Chivers, Nigel J. Robinson

**Affiliations:** 1 Department of Biosciences, Durham University, Durham, DH1 3LE, UK; 2 Department of Chemistry, Durham University, Durham, DH1 3LE, UK

**Keywords:** Metal specificity, Metalation calculator, Metal sensors, Metal availability, Magnesium, Manganese, Iron, Cobalt, Nickel, Copper, Zinc

## Abstract

Inorganic metals supplement the chemical repertoire of organic molecules, especially proteins. This requires the correct metals to associate with proteins at metalation. Protein mismetalation typically occurs when excesses of unbound metals compete for a binding site *ex vivo*. However, in biology, excesses of metal-binding sites typically compete for limiting amounts of exchangeable metals. Here, we summarise mechanisms of metal homeostasis that sustain optimal metal availabilities in biology. We describe recent progress to understand metalation by comparing the strength of metal binding to a protein versus the strength of binding to competing sites inside cells.

## Metals in proteins

Metalloproteins require metals either to impart structure, for example, to form the zinc fingers that are so prevalent in the human proteome or to assist catalysis. Of the enzymes in the protein data bank that have been structurally characterised by experiment, an estimated 47% need metals [1,2]. The distinct chemical properties of different metals add vital functionalities to catalytic sites, for example, stabilising negative charge, as Lewis acids to activate substrates, and redox-active metals serving as conduits for electrons, all in combination with the protein coordination sphere [3]. Magnesium, manganese, iron, cobalt, nickel, copper and zinc are widely used in biology. Calcium has signalling roles and is not considered here, along with molybdenum and tungsten used in some species [4]. In polluted environments, some specialised enzymes have evolved to exploit normally solely toxic metals such as cadmium, and elements such as lanthanides have been added to the known catalogue relatively recently [5,6].

Some fully folded proteins entrap metals, whereas other protein-bound metals remain labile and susceptible to exchange throughout the lifetime of a protein. This article focuses on the process of metalation, the acquisition of metals by proteins, within living organisms. More specifically, it addresses the fidelity of the metalation process that enables the elements with the required chemical properties to become bound to each individual protein. The next section highlights why bioinorganic chemistry presents a challenge to fidelity, although ultimately provides the solutions. This article presents an equilibrium thermodynamic framework for biological metalation which in turn will provide the context for understanding the magnitude of kinetic contributions. Notably, this approach alone largely explained metal specificity in the handful of systems examined to date and, in some cases, identified disparities that informed further experimentation into metal specificity, for example, into the contributions of adducts with nucleotide cofactors [7∗, 8∗∗, 9∗∗].

## Mismetalation

In the absence of steric selection, binding of common, essential, divalent metal ions to biomolecules typically follows the Irving–Williams series [4,10]. Cu^+^ predominates in the reducing environment of the cytosol and forms exceptionally tight complexes especially with thiol-containing sites [4]. Proteins are inherently flexible, especially so as they first emerge from the ribosome, and the preferred order of metal binding tends to align with this series (note reversal of the arrow after copper).

$$\begin{array}{l} {\text{Mg}}^{2+}\text{<}{\text{Mn}}^{2+}\text{<}{\text{Fe}}^{2+}\text{<}{\text{Co}}^{2+}\text{<}{\text{Ni}}^{2+}\text{<}{\text{Cu}}^{2+}\left({\text{Cu}}^{+}\right)\text{>}{\text{Zn}}^{2+}\\ \text{Weakest}\;\;\text{Tightest} \end{array}$$

For example, calculations show that all mononuclear Mg^2+^ sites in proteins bind Zn^2+^ orders of magnitude more tightly than Mg^2+^ [11,12]. In equimolar mixtures of the previous metals at surplus concentrations, proteins that require weaker-binding ions will become mismetalated with the tighter ones. The prevalence of metalloproteins generates substantial interest in understanding how biological protein metalation avoids overwhelming enzyme inactivation through mismetalation.

Binding of an aberrant metal can distort the geometry of a cognate metal site, recruit additional ligands or exploit only a subset of the native ligands. The biological challenge is to avoid tight binding of wrong ions at locations within proteins that prevent binding of the cognate metals, regardless of whether the binding sites are nonconservative. A protein can more readily discern molecules as opposed to single atoms. Thus, the challenge is simpler if preformed metal-containing cofactors bind such as haeme, siroheme, vitamin B_12_, cofactor F430 or iron-sulphur clusters. However, this shifts the challenge to the prior partitioning of the correct metal during synthesis of the cofactors followed by specific protein–protein interactions if there is a cofactor delivery protein [13,14]. Metallochaperones and chelatases supply metals directly to some proteins [15∗, 16∗∗, 17, 18]. Again, selective protein–protein interactions assist specificity, but the residual challenge remains the prior partitioning of the correct metal onto the delivery or insertion protein.

Mismetalation does occur in biology and may be more widespread than currently documented [19∗∗, 20, 21, 22, 23∗]. Mismetalation coincides with altered metal bioavailability [19,20]. Two cupins, manganese cupin A (MncA) and copper cupin A (CucA), illustrate the importance of metal bioavailability to protein metal specificity [24]. These two proteins share common folds and common metal-binding ligands, but in the outer periplasmic compartment of a cyanobacterium, MncA is associated with weak-binding Mn^2+^, whereas CucA contains tight-binding Cu^2+^. Metalation and folding *in vitro* in the presence of equimolar concentrations of metals lead MncA to select and entrap either Cu^+^, Cu^2+^ or Zn^2+^ in preference to Mn^2+^ [24]. A nascent site along the MncA-folding pathway appears to obey the Irving–Williams series. Importantly, *in vivo* synthesis and export of the copper-protein CucA to the periplasm use the standard general secretion Sec-pathway for unfolded proteins, but MncA uses the less common twin-arginine translocation Tat pathway for prefolded proteins. MncA thus acquires and entraps Mn^2+^ in the cytosol before export. The biological environment, in this example, the cytosol, must restrict disfavourable competition from tighter-binding metals such as Cu^+^, Cu^2+^ or Zn^2+^ at MncA folding. This directs our attention towards the biological control of metal availability.

## The components of biological metallostasis

**Figure 1:**
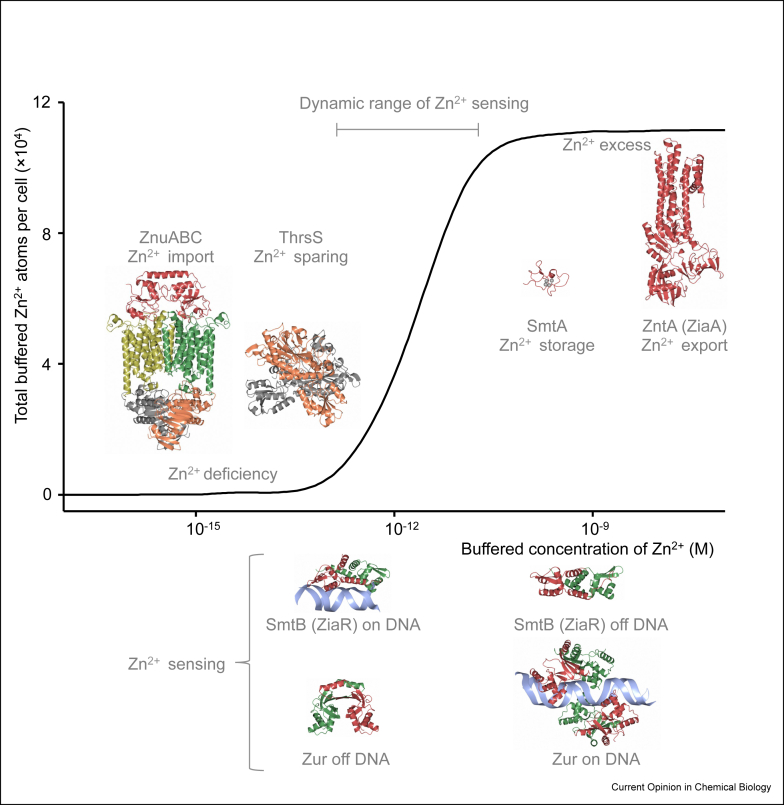
**The biological control of metal availability**. When intracellular metal (such as Zn^2+^ in a cyanobacterium) becomes depleted, importers (such as zinc uptake (ZnuABC) represented by PDB 4FI3) acquire more, and cells switch to proteins that reduce demand (metal sparing) (exemplified by *Anabaena* threonine aminoacyl-tRNA synthetase (ThrRS) [49], represented by PDB 6L2P). When intracellular metal becomes surplus, exporters (such as zinc translocating P-type ATPase (ZntA) represented by PDB 4UMV) and storage proteins (such as Synechococcus metallothionein (SmtA) PDB 1JJD) remove excess. Sensors (such as zinc uptake regulator (Zur) represented by PDB 4MTD, 3MWM and Synechococcus metallationein regulator (SmtB) PDB 1R23 and represented by PDB 4OMY) detect the transition from deficiency to surplus to enable adaptation by regulating production of import, export, storage and sparing mechanisms. Modelling of a hypothetical Zn^2+^ buffer is adapted from the work of Osman et al. [8].

A catalogue of metal importers, metal exporters, metal storage proteins and metal sparing mechanisms enable organisms to adapt to changes in metal supply to maintain intracellular (and some intercellular albeit not discussed here) metal availabilities [23,25, 26, 27, 28, 29, 30]. Different complements of these systems exist for different metals and vary between cell types and organisms [23,25]. Their activities optimise metal bioavailability by acquiring more of a metal or switching to alternative metals to reduce demand when there is deficiency, or by exporting, storing or using more when a metal is in surplus (Figure 1). This further focuses our attention towards the mechanisms that detect the deficiencies and surpluses of each metal ion.

Metal-responsive changes in the transcription of genes encoding proteins of metallostasis are widely documented [1], and numerous metal-sensing regulators of gene transcription have been described [1]. DNA-binding metal-sensing transcriptional regulators have been especially well-characterised in bacteria [23,31]. On binding metal ions, these transcriptional regulators undergo change in structure and/or dynamics to alter DNA binding [31]. The allosteric mechanisms of cytosolic metal sensors can act in several ways. Metal binding may weaken DNA binding, exemplified by derepressors of metal export (such as ZiaR in Figure 1) [31,32], tighten DNA binding, exemplified by corepressors of metal import (such as Zur in Figure 1) [23,31] or distort DNA to enable transcription, exemplified by a subset of coactivators of metal export (such as ZntR) [31,33]. By whichever mechanism, these sensors somehow recognise intracellular metal availability.

## Associative metalation and available metal

What is intracellular available metal? Metal (M) transfer between biomolecules (X and P) may be dissociative or associative via adducts (PMX), and the current opinion is that the latter dominates metalation in biology.

DissociativeP+MX↔P+M+X↔PM+X

AssociativeP+MX↔PMX↔PM+X

**Figure 2:**
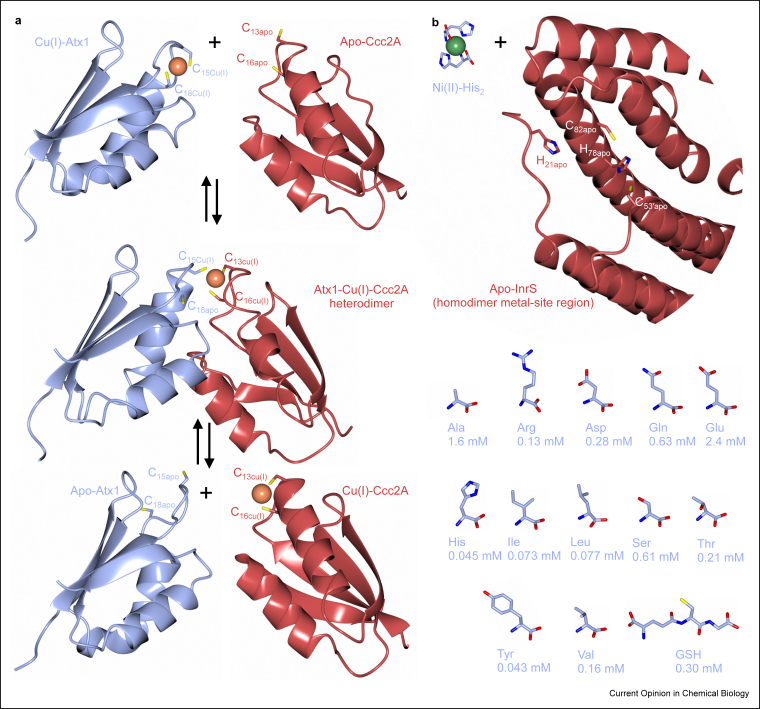
**Associative ligand exchange**. (**a)**. Cu^+^ transfer by ligand exchange from a metallochaperone (antioxidant protein (Atx1) PDB 1FD8, 1FES) to its cognate cupro-protein (copper transporting ATPase soluble domain 2 (Ccc2A) PDB 1FVQ, 1FVS) via a heteroprotein complex (PDB 2GGP). (**b)**. Metalation of InrS (PDB 5FMN) depends on its ability to compete for Ni^2+^ with buffering histidine molecules within a simplified cytosol-like mixture [7]. It is proposed that associative metal transfer by ligand exchange also occurs from buffering small molecule complexes, such as Ni^2+^histidine_2_, to a protein such as internal nickel responsive sensor (InrS) [7].

Associative metal transfer by ligand exchange has been extensively described and visualised in the transfer of Cu^+^ from metallochaperones to some cuproproteins [16] (Figure 2a), but delivery proteins are not known for most metalloproteins. The concentrations of Zn^2+^ that trigger two *Escherichia coli* metal sensors in an *in vitro* transcription system are in the femtomolar range [34]. In contrast, the total amount of Zn^2+^ divided by the *E. coli* cell volume would suggest millimolar Zn^2+^ concentrations [34]. The difference between these millimolar and femtomolar values reflects the vast number of cellular Zn^2+^-binding sites. This suggests that Zn^2+^ proteins acquire metal either improbably via the dissociative route from a negligible source (one atom per cell volume equates to nanomolar) that is fleetingly available in one millionth of cells or from specialised delivery proteins [15,34]. However, the second option demands the discovery of more delivery proteins and then revisits questions about how delivery proteins acquire the correct metal. Studies of the internal nickel-responsive sensor (InrS) have highlighted a third option: metalation via associative ligand exchange but not necessarily from dedicated delivery proteins.

**Figure 3:**
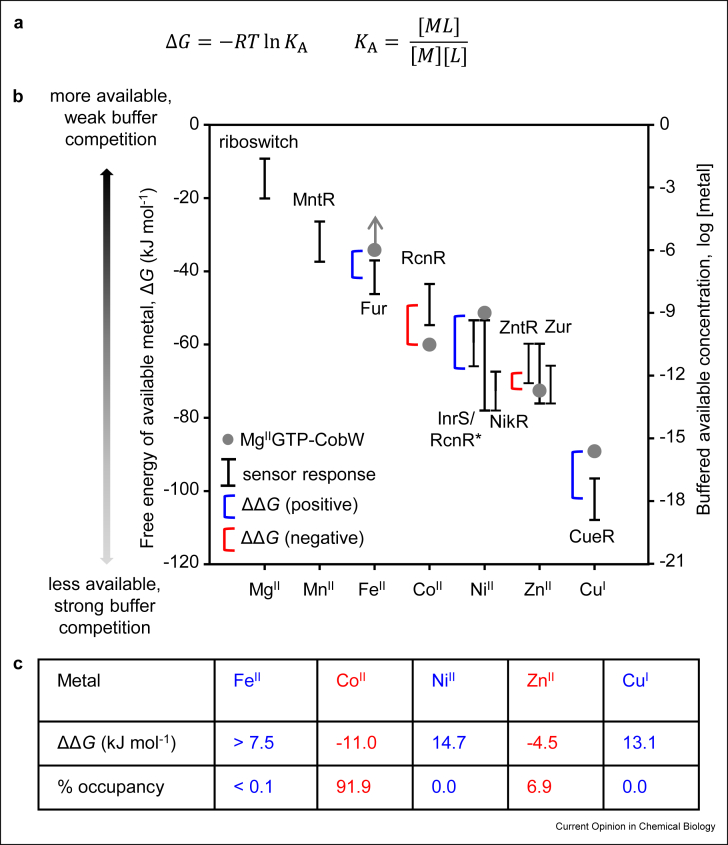
**Metal availabilities read-out from metal sensors and used to calculate metalation**. (**a)**. Standard relationships between free energy change (Δ*G*) and association constant (*K*_A_), then *K*_A_ with metal (M), ligand (L) and complex (ML) concentrations (R = molar gas constant, T = temperature in kelvin). (**b).** Cells maintain metal availabilities to the inverse of the Irving–Williams series [8,10]. Bars reflect 10%–90% of the range of each metal sensor shown as free energy for forming a metal complex that would be 50% metalated (left axis) at the respective metal concentration (right axis). Data adapted from *Salmonella* sensors [8], plus Mg^2+^ riboswitch [50], with Ni^2+^ detection also calculated using metal and DNA affinities (*nrsD* promoter) for cyanobacterial InrS [7], to simulate high Ni^2+^ detection by *Salmonella* RcnR (hence RcnR∗). Circles show Δ*G* for forming metal complexes with Co^2+^ chaperone for B_12_, Mg^2+^GTP-CobW (left axis) and metal concentration where Mg^2+^GTP-CobW is 50% saturated (right axis). Arrow indicates that only a limiting free energy was determined, Δ*G* may be more positive. (**c).** Differences in free energy for metal complex formation with Mg^2+^GTP-CobW versus the intracellular milieu (ΔΔ*G*) in an ideal cell where sensors are at their midpoints identify favourable (red, also on panel b) or unfavourable (blue) thermodynamic gradients for metalation. A metalation calculator compares such values to calculate *in vivo* metal occupancies [9].

Mutants of InrS with weakened affinities for Ni^2+^ lost the ability to regulate a target gene nickel response system (*nrsD*) in response to Ni^2+^ inside viable engineered cells, even though DNA binding remained allosterically coupled to Ni^2+^ binding *in vitro* [7]. Concentrations of the most abundant amino acids plus glutathione were measured in the same cells, and then InrS was competed for Ni^2+^ against a matched synthetic buffer (Figure 2b). Wild-type protein readily acquired Ni^2+^ from the synthetic buffer, whereas the variants did not, and the dominant-competing molecule in this system was free histidine [7]. Associative ligand exchange is proposed for Ni^2+^ transfer to InrS but from small molecule complexes such as Ni^2+^ histidine that act as metal buffers (Figure 2). Some of the predominant molecules that bind and buffer selected metal ions in the cytosol of different organisms include glutathione [35,36], bacillithiol in Firmicute bacteria [37], amino acids [38,39] and specialised proteins such as metallothioneins [40]. The almost nonexistent concentrations of unbound hydrated metals inside living cells thus become irrelevant for metalation with one caveat: these values reflect how tightly the intracellular milieu binds to exchangeable available metals (Figure 3a and b).

## Intracellular metal availability follows the inverse of the Irving–Williams series

There has been a long-standing prediction that cytoplasmic free metal concentrations are the inverse of the Irving–Williams stability constant series [4]. Here, we interpret free metal as hydrated metal and understand that these concentrations report how tightly exchangeable available metal is bound. If the prediction is correct, there will only have been sufficient selection pressure for proteins to discern the correct metal under these availabilities, regardless of whether greater discrimination could have evolved in different cellular conditions. To test the prediction, the tuning of a set of bacterial metal sensors (from *Salmonella entrica* serovar Typhimurium) was determined [8]. The sensors included metal-dependent corepressors responsive to Mn^2+^ (MntR), Fe^2+^ (Fur), Ni^2+^ (NikR), Zn^2+^ (Zur), activators responsive to Cu^+^ (CueR), Zn^2+^ (ZntR) and a derepressor responsive to Co^2+^ and Ni^2+^ (RcnR) [8,31].

The sensitivity to metal of a DNA-binding metal sensor is a function of its affinity (for a metal and for its target promoter DNA), its allosteric mechanism [41], and its abundance especially if this changes with metal availability [8]. These combine to tune a sensor to respond within the dynamic range of availabilities over which the cognate metal fluctuates inside viable cells, thereby matching sensitivity to variable access to metal [8,42]. A thermodynamic cycle couples DNA binding and release to metal binding and release [43,44]. For each *Salmonella* metal sensor *K*_metal_ of the allosteric site, *K*_promoterDNA_ (for apo- and holo-sensor), the number of sensor molecules per cell (in the presence and absence of elevated metal) and number of promoter targets were determined [8]. The fraction of promoter DNA occupied by each sensor was then calculated as a function of metal availability [8]. The respective thermodynamic cycle coupling DNA binding and release with metal binding and release can be resolved mathematically provided metal binding to the sensor does not alter buffered metal availability. Figure 3b represents the availabilities over which sensors respond (from 10% to 90% of the full responses), shown as buffered concentrations (right y-axis) and as free energies for forming metal complexes that would be 50% saturated at the respective buffered concentrations (left y-axis). These data establish that the tuning of metal sensors is set to maintain metal availabilities to the inverse of the Irving–Williams series (Figure 3b). The long-standing prediction is correct.

## A biological metalation calculator

Figure 3b reveals the magnitude of competition from exchangeable binding sites in the cytosol for each metal. By comparing the metal affinities of proteins, also expressed as free energies for complex formation, with these values, *in vivo* metalation becomes fathomable [8]. For example, a GTPase involved in Co^2+^ supply for vitamin B_12_ biosynthesis, cobalamin operon gene W (CobW), is shown to outcompete the cytosol for Co^2+^ and for Zn^2+^ when bound to Mg^2+^GTP (and when the sensors are at their midpoints of their dynamic ranges in idealised cells) [9]. Figure 3b thus shows that the thermodynamic gradient for ligand exchange to Mg^2+^GTPCobW is favourable (negative shown in red) for Co^2+^ and for Zn^2+^ but unfavourable (positive shown in blue) for other metals even though the protein binds Cu^+^most tightly. The free energy difference is greater for Co^2+^ than Zn^2+^ (Figure 3c). Thus, the thermodynamic gradient for ligand exchange is steepest for Co^2+^, and so the protein preferably binds Co^2+^. These concepts have enabled production of a metalation calculator that outputs intracellular metalation for a protein of known metal affinities based on the relative magnitudes of competition for each metal with exchangeable sites as determined from the responses of metal sensors [9].

The metalation calculator has also shown that CobW will release Co^2+^ (or Zn^2+^) on nucleotide hydrolysis [9]. The ranges for each sensor shown in Figure 3b reflect differing availabilities at different degrees of buffer saturation with metal. The position of each metal sensor within its range in a conditional cell culture can be determined from isolated transcripts, for example, by quantitative polymerase chain reaction (qPCR) [9]. This can therefore read-out change in intracellular metal availability with growth under different metal conditions. Inputting such values into the calculator reveals that in low cobalt media Mg^2+^GTPCobW becomes mismetalated with Zn^2+^ because the thermodynamic gradient becomes more shallow for Co^2+^ than Zn^2+^ [9]. Notably, the calculated metalation of Mg^2+^GTPCobW with Co^2+^ under different growth conditions matches *cobW*-dependent synthesis of vitamin B_12_ [9]. The calculator also shows that Mg^2+^GTP-bound forms of two related GTPases, YeiR and YjiA, preferentially acquire Zn^2+^ in ideal cells, not because they have tighter affinities for Zn^2+^ relative to Mg^2+^GTPCobW, but rather, because they possess weaker affinities for Co^2+^ [9]. Unlike Mg^2+^GTPCobW, competition between these proteins and the cytosol for Co^2+^ is insufficient to displace Zn^2+^.

## Prospects

To understand the specificity of metalation in biological systems, we now appreciate that it is necessary to know the magnitude of competition from other sites. Here, we see this achieved by calibrating the DNA-binding metal sensors of *Salmonella* [8] and by inference *E. coli* with its similar complement of metal sensors [9]. There is a necessity to discover how much the tuning of DNA-binding metal sensors varies across a diversity of bacterial species. A number of chemical and biological probes respond to metals *in vivo* [45, 46, 47, 48]. These probes might provide an alternative route to measure available metal concentrations inside cells and hence to determine the free energies of exchangeable available metal. In turn, this could reveal the magnitude of competition for each metal from intracellular binding sites in a multitude of other biological systems. Where a single defined molecule dominates the intracellular buffering and binding sites for a particular metal, the magnitude of competition for that metal should become calculable from the metal affinities of that molecule.

In the future, thermodynamic frameworks as in Figure 3b and as used in the metalation calculator [9] should reveal the magnitudes of the contributions of additional mechanisms to protein metal speciation such as the formation of adducts exemplified here by associations with Mg^2+^GTP,; kinetic trapping after folding, redox changes, steric selection via the formation of binuclear sites and more. The need to replace aspects of manufacturing based on fossil fuels with something more sustainable driven by biological catalysts, coupled with the high prevalence of metalloenzymes, creates an imperative to make biological protein metalation accessible and exploitable. Metalation calculators need to be useable by technologists with expertise outside biological chemistry.

## Declaration of competing interest

The authors declare that they have no known competing financial interests or personal relationships that could have appeared to influence the work reported in this article.

## References

[1] Waldron K.J., Rutherford J.C., Ford D., Robinson N.J. (2009). Metalloproteins and metal sensing. Nature.

[2] Valasatava Y., Rosato A., Furnham N., Thornton J.M., Andreini C. (2018). To what extent do structural changes in catalytic metal sites affect enzyme function?. J Inorg Biochem.

[3] Fernandes H.S., Teixeira C.S.S., Sousa S.F., Cerqueira N. (2019). Formation of unstable and very reactive chemical species catalyzed by metalloenzymes: a mechanistic overview. Molecules.

[4] Fraústo da Silva J.J.R., Williams R.J.P. (1991).

[5] Cotruvo J.A., Featherston E.R., Mattocks J.A., Ho J.V., Laremore T.N., Lanmodulin (2018). A highly selective lanthanide-binding protein from a lanthanide-utilizing bacterium. J Am Chem Soc.

[6] Featherston E.R., Cotruvo J.A. (2021). The biochemistry of lanthanide acquisition, trafficking, and utilization. Biochim Biophys Acta Mol Cell Res.

[bib7] Foster A.W., Pernil R., Patterson C.J., Scott A.J.P., Pålsson L.O., Pal R., Cummins I., Chivers P.T., Pohl E., Robinson N.J. (2017). A tight tunable range for Ni(II) sensing and buffering in cells. Nat Chem Biol.

[bib8] Osman D., Martini M.A., Foster A.W., Chen J., Scott A.J.P., Morton R.J., Steed J.W., Lurie-Luke E., Huggins T.G., Lawrence A.D. (2019). Bacterial sensors define intracellular free energies for correct enzyme metalation. Nat Chem Biol.

[bib9] Young T.R., Martini M.A., Foster A.W., Glasfeld A., Osman D., Morton R.J., Deery E., Warren M.J., Robinson N.J. (2021). Calculating metalation in cells reveals CobW acquires Co(II) for vitamin B(12) biosynthesis while related proteins prefer Zn(II). Nat Commun.

[bib10] Irving H., Williams R.J.P. (1948). Order of stability of metal complexes. Nature.

[bib11] Dudev T., Lim C. (2001). Metal selectivity in metalloproteins: Zn2+ vs Mg2+. J Phys Chem *B*.

[12] Yang T.-Y., Dudev T., Lim C. (2008). Mononuclear versus binuclear metal-binding sites:metal -BindingAffinity and selectivity from PDB survey and DFT/CDMCalculations. J Am Chem Soc.

[13] Galmozzi A., Kok B.P., Kim A.S., Montenegro-Burke J.R., Lee J.Y., Spreafico R., Mosure S., Albert V., Cintron-Colon R., Godio C. (2019). PGRMC2 is an intracellular haem chaperone critical for adipocyte function. Nature.

[14] Patel S.J., Protchenko O., Shakoury-Elizeh M., Baratz E., Jadhav S., Philpott C.C. (2021). The iron chaperone and nucleic acid-binding activities of poly(rC)-binding protein 1 are separable and independently essential. Proc Natl Acad Sci U S A.

[bib15] Pufahl R.A., Singer C.P., Peariso K.L., Lin S.J., Schmidt P.J., Fahrni C.J., Culotta V.C., Penner-Hahn J.E., O'Halloran T.V. (1997). Metal ion chaperone function of the soluble Cu(I) receptor Atx1. Science.

[bib16] Banci L., Bertini I., Cantini F., Felli I.C., Gonnelli L., Hadjiliadis N., Pierattelli R., Rosato A., Voulgaris P. (2006). The Atx1-Ccc2 complex is a metal-mediated protein-protein interaction. Nat Chem Biol.

[17] Bryant D.A., Hunter C.N., Warren M.J. (2020). Biosynthesis of the modified tetrapyrroles-the pigments of life. J Biol Chem.

[18] Edmonds K.A., Jordan M.R., Giedroc D.P. (2021). COG0523 proteins: a functionally diverse family of transition metal-regulated G3E P-loop GTP hydrolases from bacteria to man. Metall.

[bib19] Privalle C.T., Fridovich I. (1992). Transcriptional and maturational effects of manganese and iron on the biosynthesis of manganese-superoxide dismutase in Escherichia coli. J Biol Chem.

[20] Imlay J.A. (2014). The mismetallation of enzymes during oxidative stress. J Biol Chem.

[21] Ranquet C., Ollagnier-de-Choudens S., Loiseau L., Barras F., Fontecave M. (2007). Cobalt stress in Escherichia coli. The effect on the iron-sulfur proteins. J Biol Chem.

[22] Macomber L., Elsey S.P., Hausinger R.P. (2011). Fructose-1,6-bisphosphate aldolase (class II) is the primary site of nickel toxicity in Escherichia coli. Mol Microbiol.

[bib23] Chandrangsu P., Rensing C., Helmann J.D. (2017). Metal homeostasis and resistance in bacteria. Nat Rev Microbiol.

[bib24] Tottey S., Waldron K.J., Firbank S.J., Reale B., Bessant C., Sato K., Cheek T.R., Gray J., Banfield M.J., Dennison C. (2008). Protein-folding location can regulate manganese-binding versus copper- or zinc-binding. Nature.

[25] Ma Z., Jacobsen F.E., Giedroc D.P. (2009). Coordination chemistry of bacterial metal transport and sensing. Chem Rev.

[26] Lonergan Z.R., Skaar E.P. (2019). Nutrient zinc at the host-pathogen interface. Trends Biochem Sci.

[27] Chareyre S., Mandin P. (2018). Bacterial iron homeostasis regulation by sRNAs. Microbiol Spectr.

[28] Giachino A., Waldron K.J. (2020). Copper tolerance in bacteria requires the activation of multiple accessory pathways. Mol Microbiol.

[29] Merchant S.S., Schmollinger S., Strenkert D., Moseley J.L., Blaby-Haas C.E. (2020). From economy to luxury: copper homeostasis in Chlamydomonas and other algae. Biochim Biophys Acta Mol Cell Res.

[30] Bradley J.M., Svistunenko D.A., Wilson M.T., Hemmings A.M., Moore G.R., Le Brun N.E. (2020). Bacterial iron detoxification at the molecular level. J Biol Chem.

[31] Baksh K.A., Zamble D.B. (2020). Allosteric control of metal-responsive transcriptional regulators in bacteria. J Biol Chem.

[32] Morby A.P., Turner J.S., Huckle J.W., Robinson N.J. (1993). SmtB is a metal-dependent repressor of the cyanobacterial metallothionein gene smtA: identification of a Zn inhibited DNA-protein complex. Nucleic Acids Res.

[33] Fang C., Philips S.J., Wu X., Chen K., Shi J., Shen L., Xu J., Feng Y., O'Halloran T.V., Zhang Y. (2021). CueR activates transcription through a DNA distortion mechanism. Nat Chem Biol.

[bib34] Outten C.E., O'Halloran T.V. (2001). Femtomolar sensitivity of metalloregulatory proteins controlling zinc homeostasis. Science.

[35] Stewart L.J., Ong C.Y., Zhang M.M., Brouwer S., McIntyre L., Davies M.R., Walker M.J., McEwan A.G., Waldron K.J., Djoko K.Y. (2020). Role of glutathione in buffering excess intracellular copper in Streptococcus pyogenes. mBio.

[36] Hider R, Aviles MV, Chen YL, Latunde-Dada GO: The role of GSH in intracellular iron trafficking. Int J Mol Sci 2021, 22. 10.3390/ijms22031278 PMC7865746 33525417

[37] Ma Z., Chandrangsu P., Helmann T.C., Romsang A., Gaballa A., Helmann J.D. (2014). Bacillithiol is a major buffer of the labile zinc pool in Bacillus subtilis. Mol Microbiol.

[38] Jordan M.R., Wang J., Weiss A., Skaar E.P., Capdevila D.A., Giedroc D.P. (2019). Mechanistic insights into the metal-dependent activation of Zn(II)-Dependent metallochaperones. Inorg Chem.

[39] Brawley H.N., Lindahl P.A. (2021). Low-molecular-mass labile metal pools in Escherichia coli: advances using chromatography and mass spectrometry. J Biol Inorg Chem.

[40] Zeng J., Vallee B.L., Kägi J.H. (1991). Zinc transfer from transcription factor IIIA fingers to thionein clusters. Proc Natl Acad Sci U S A.

[bib41] Cavet J.S., Meng W., Pennella M.A., Appelhoff R.J., Giedroc D.P., Robinson N.J. (2002). A nickel-cobalt-sensing ArsR-SmtB family repressor. Contributions of cytosol and effector binding sites to metal selectivity. J Biol Chem.

[42] Osman D., Foster A.W., Chen J., Svedaite K., Steed J.W., Lurie-Luke E., Huggins T.G., Robinson N.J. (2017). Fine control of metal concentrations is necessary for cells to discern zinc from cobalt. Nat Commun.

[bib43] VanZile M.L., Chen X., Giedroc D.P. (2002). Allosteric negative regulation of smt O/P binding of the zinc sensor, SmtB, by metal ions: a coupled equilibrium analysis. Biochemistry.

[44] Capdevila D.A., Huerta F., Edmonds K.A., Le M.T., Wu H., Giedroc D.P. (2018). Tuning site-specific dynamics to drive allosteric activation in a pneumococcal zinc uptake regulator. Elife.

[45] Pratt E.P.S., Damon L.J., Anson K.J., Palmer A.E. (2021). Tools and techniques for illuminating the cell biology of zinc. Biochim Biophys Acta Mol Cell Res.

[46] Chung C.Y., Posimo J.M., Lee S., Tsang T., Davis J.M., Brady D.C., Chang C.J. (2019). Activity-based ratiometric FRET probe reveals oncogene-driven changes in labile copper pools induced by altered glutathione metabolism. Proc Natl Acad Sci U S A.

[47] Fahrni C.J. (2013). Synthetic fluorescent probes for monovalent copper. Curr Opin Chem Biol.

[48] Zastrow M.L., Huang Z., Lippard S.J. (2020). HaloTag-based hybrid targetable and ratiometric sensors for intracellular zinc. ACS Chem Biol.

[49] Rubio M., Napolitano M., Ochoa de Alda J.A., Santamaría-Gómez J., Patterson C.J., Foster A.W., Bru-Martínez R., Robinson N.J., Luque I. (2015). Trans-oligomerization of duplicated aminoacyl-tRNA synthetases maintains genetic code fidelity under stress. Nucleic Acids Res.

[50] Dann C.E., Wakeman C.A., Sieling C.L., Baker S.C., Irnov I., Winkler W.C. (2007). Structure and mechanism of a metal-sensing regulatory RNA. Cell.

